# A Rare Case of Cardiac Tamponade in a Patient With Anti-RNA Polymerase III Scleroderma

**DOI:** 10.7759/cureus.38533

**Published:** 2023-05-04

**Authors:** Artur Schneider, Nikita Jhawar, Lisa Balistreri, Razvan Chirila, Florentina Berianu

**Affiliations:** 1 Internal Medicine, Mayo Clinic, Jacksonville, USA; 2 Rheumatology, Mayo Clinic, Jacksonville, USA

**Keywords:** anti-rna polymerase 3 autoantibody, dyspnea, pericardial effusion, cardiac tamponade, scleroderma

## Abstract

Scleroderma is a chronic, progressive autoimmune disease that often presents with multiorgan involvement. Cardiac manifestations are common and include microvascular coronary artery disease, conduction abnormalities, autonomic insufficiency, and pericardial effusions. Although rare, pericardial effusions may progress and lead to cardiac tamponade. Patients diagnosed with scleroderma can be further prognosticated based on the presence of serologic scleroderma-specific antibodies. The anti-RNA polymerase III autoantibody (anti-RNAP3) is associated with an aggressive subtype of scleroderma. Looking at the current literature, no association has been reported between anti-RNAP3 and the development of cardiac tamponade in patients with underlying scleroderma. We discuss a unique case of a patient with scleroderma who was found to be anti-RNAP3 positive and signs of cardiac tamponade. This case illustrates the importance of an expeditious diagnosis and timely interventions to treat cardiac tamponade. Additionally, we share a rare but important association between anti-RNAP3 and the formation of tamponade physiology in scleroderma.

## Introduction

Scleroderma is a chronic autoimmune disease that targets multiple organs. As seen in most autoimmune diseases, scleroderma more frequently affects women, especially in the fifth decade of life. The extent of involvement varies and can manifest broadly from limited disease, affecting one organ system, to diffuse cutaneous disease with multiorgan involvement.

Cardiac manifestations in scleroderma are often not recognized until late in the disease course as they initially often present with vague, nonspecific symptoms, but can affect all parts of the heart, including the myocardium, pericardium, and conduction system. The pathogenesis involves vascular changes, activation of the innate and adaptive immune system, as well as fibrosis that leads to scarring and organ failure [1]. In cardiac involvement, in particular, the physiology is best explained by a primary process affecting the heart or secondary to pulmonary hypertension, interstitial lung disease, or scleroderma renal crisis [1]. Pericardial effusions can be the first manifestation of the disease, especially in patients with diffuse cutaneous involvement [2]. However, pericardial effusions rarely cause tamponade physiology in scleroderma patients because of their slow progression and the heart’s ability to adapt [3]. Here, we describe a unique case of diffuse scleroderma in a patient who presented with dyspnea and imaging findings suggestive of a large pericardial effusion complicated by tamponade physiology.

## Case presentation

**Figure 1:**
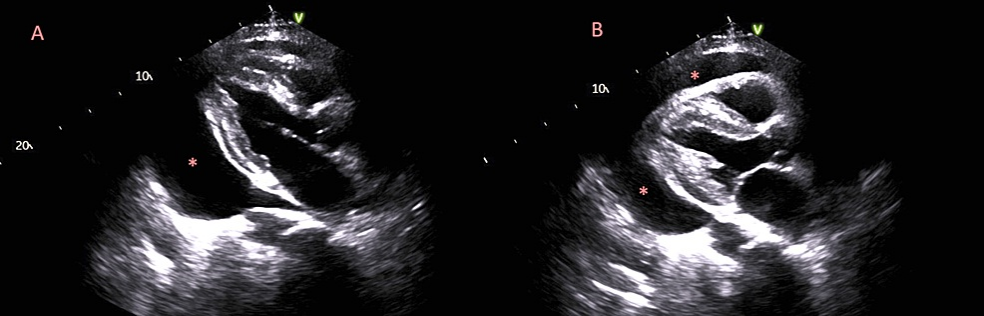
Echocardiogram showing tamponade physiology. Echocardiogram demonstrating pericardial effusion with findings suggesting tamponade physiology. A: Large pericardial effusion between the myocardium and pericardial sac (red asterisk) with associated diastolic right ventricular collapse. B: Swinging heart physiology illustrated by changing pericardial effusion diameters and location (red asterisks).

A 58-year-old male with a past medical history of known RNA polymerase III (RNAP3)-positive scleroderma with skin involvement, Raynaud’s phenomenon, and hypertension presented to the emergency department from his outpatient rheumatology appointment complaining of dyspnea. In the outpatient clinic, an echocardiogram showed an ejection fraction of 43%, no regional wall motion abnormalities, moderately enlarged right ventricular chamber size with reduced systolic function, and moderate-to-severe tricuspid regurgitation. Furthermore, the echocardiogram revealed a swinging heart pericardial effusion and respiratory variation in left ventricular and right ventricular (LV/RV) dimensions, all of which suggested the presence of tamponade (Figure 1). Aside from worsening dyspnea for several weeks, the patient’s review of systems was negative without any mention of lightheadedness, dizziness, chest pain, palpitations, fever, and chills. At the time of his presentation, the patient was not on immunosuppressive therapy but was receiving topical therapy for his diffuse skin involvement, including calcipotriene 0.005% cream, clobetasol 0.05% ointment, and collagenase 250 U/g ointment. In addition, the patient was taking tamsulosin 0.4 mg daily, valsartan 40 mg daily, and naproxen 250 mg daily as needed for pain.

On physical examination, the patient appeared well-developed and was not in apparent distress. Vital signs revealed a temperature of 36.6°C, heart rate of 81 beats per minute, respiratory rate of 24 breaths per minute, blood pressure of 127/56 mmHg, and oxygen saturation of 99%. A pulmonary examination revealed no abnormalities. A cardiovascular examination showed regular rate and rhythm, decreased S1 and S2 sounds, and jugular venous pressure elevation at 11 cm. Complete blood count and renal function panel were within normal limits. Sedimentation rate was 5 mm/hour (0-22 mm/hour), C-reactive protein was 3.8 mg/L (≤8.0 mg/L), antinuclear antibody was 8.6 U (≤1.0 U), anti-RNAP3 was 135.9 U (<20.0 U), SS-A/Ro antibody was >8.0 U (<1.0 U), aldolase was 5.0 U/L (<7.7 U/L). Centromere antibody, SS-B/La antibody, Sm antibody, RNP antibody, Scl 70 antibody, and Jo 1 antibody were all negative. The N-terminal pro-brain natriuretic peptide was 901 pg/mL (≤88 pg/mL), and the electrocardiogram (EKG) showed normal sinus rhythm with low voltage QRS. Thyroid-stimulating hormone (TSH) was not measured.

The patient was admitted to the hospital for a pericardiocentesis via a pericardial drain and underwent a right heart catheterization (RHC). The pericardial drain was placed, and 1.5 L of yellow serous fluid was drained. Pericardial fluid analysis revealed lactate dehydrogenase (LDH) of 147 U/L (>0.6 suggestive of exudative effusions in pleural effusions; not well established in pericardial effusions). Although an increased number of mesothelial cells were noted, no blasts or malignant cells were seen. Serum LDH was not measured. Gram stain, fungal smear, and acid-fast bacilli (AFB) were negative. RHC showed an elevated pulmonary capillary wedge pressure (23 mmHg; reference range = 4-12 mmHg), mean pulmonary artery pressure of 54 mmHg (<20 mmHg), pulmonary vascular resistance of 7.45 WU (<3 WU), suggestive of pulmonary hypertension. The patient reported a significant improvement in dyspnea after the pericardiocentesis, and the post-procedure echocardiogram showed a stable posterior effusion with no evidence of tamponade physiology. The rheumatology consulting service recommended a seven-day course of prednisone 20 mg oral daily, followed by a taper of a 5 mg daily decrease every week. It was noted that higher doses of prednisone should be avoided due to the risk of potential scleroderma renal crisis. Due to the patient’s clinical improvement and minimal re-accumulation of fluid in the drain, the pericardial drain was removed, and the patient was discharged.

A repeat echocardiogram during the patient’s post-hospitalization follow-up one week later showed a recurrence of pericardial effusion without overt signs of tamponade. The patient was re-admitted for recurrent pericardial effusion and underwent another pericardiocentesis. Serologic testing showed an elevated CRP of 78.0 mg/L (reference <8), suggestive of continued inflammation. TSH was elevated at 5.7 mlU/L (0.3-4.2 mlU/L), and the thyroperoxidase antibody was negative. Given the association with malignancies, prostate-specific antigen, serum protein electrophoresis, and urine protein electrophoresis were measured but were within normal limits and did not show findings of monoclonal proteins. Hepatitis B and C were negative. The rheumatology consultant service suggested starting mycophenolate mofetil 500 mg twice daily with the goal to increase the dose to 1 g twice daily as tolerated while continuing to wean prednisone, as it was stated to be a first-line therapy in patients with systemic sclerosis who are at risk for progressive interstitial lung disease. Due to concerns for scleroderma renal crisis with ongoing prednisone use, close monitoring while weaning prednisone was recommended. In addition, the patient was started on high-dose indomethacin, omeprazole, and colchicine. A repeat echocardiogram after the pericardiocentesis showed only a small residual effusion which, along with decreasing CRP levels, indicated an improvement in clinical status, and the patient was safely discharged.

A few weeks after discharge, the patient developed recurrent, worsening effusions and eventually underwent subxiphoid pericardial window placement.

## Discussion

This case highlights the importance of recognizing cardiac manifestations of scleroderma and maintaining a broad differential in patients with vague symptoms such as dyspnea. To our knowledge, this is the first time an association between anti-RNAP3 seropositivity and cardiac tamponade formation has been suggested. While some cardiac manifestations may not be identified before an autopsy, others may be life-threatening and represent the first presenting complaint [1,2]. The literature suggests that up to two-thirds of patients suffering from scleroderma have cardiac involvement [4].

Initial evaluation of pericardial effusion includes a 12-lead EKG and transthoracic echocardiogram. Patients with acute pericarditis may present with diffuse ST elevations and PR depressions on EKG, while patients with large pericardial effusions may have EKG findings showing QRS alternans. Echocardiographic findings may be normal or may show a pericardial effusion or evidence of constrictive pericarditis. Dissociation of intrathoracic and intracardiac pressures along with enhanced ventricular interaction are echocardiographic findings suggestive of constrictive pericarditis [5].

This case further highlights the importance of serologic testing for autoantibodies in patients presenting with scleroderma. A review of the literature suggests that patients who are anti-RNAP3 positive often suffer from a clinically more aggressive and diffuse phenotype. Contrary to the two most common autoantibodies found in scleroderma, anti-topoisomerase I and anti-centromere, anti-RNAP3 results in a higher risk for scleroderma renal crisis, malignancy, contractures, and rapidly progressing skin involvement [6].

Cardiac involvement in scleroderma includes microvascular coronary artery disease from poor circulation and vascular reactivity, conduction defects, and tachyarrhythmias secondary to microvascular damage and fibrosis, as well as autonomic insufficiency which by itself has been shown to predict increased mortality and risk of sudden cardiac death [7-9]. Scleroderma patients present with heart failure due to physiologic dysfunction and fibrosis at multiple anatomical sites in the heart [10]. Pericardial effusions are reported at a high rate in patients suffering from scleroderma, especially if diffuse involvement is present. However, pericardial effusions in scleroderma are often not associated with clinically measurable cardiac dysfunction [11]. Interestingly, to tie back to the importance of serology, rapid and diffuse scleroderma involvement has been more commonly associated with anti-RNAP3 autoantibodies. This holds true even when patients are positive for other autoantibodies, suggesting the clinical importance of having a positive anti-RNAP3 [5]. A review of the current literature shows that anti-RNAP3 autoantibodies are associated with pericardial effusions [12]. However, tamponade physiology in anti-RNAP3-positive patients has not been described. Although a more thorough initial workup possibly including a pericardial biopsy would be helpful to hone in on the true etiology of the recurrent pericardial effusions in this patient, one could question whether or not there is an underlying association between anti-RNAP3 seropositivity and the formation of cardiac tamponade and recurrent pericardial effusions.

The true pathophysiology of primary pericardial disease in scleroderma remains unknown, and treatment is similar to that of pericardial effusion in the absence of underlying scleroderma [13]. Treatment options include observation, pericardial drainage for large and symptomatic effusions, non-steroidal anti-inflammatory drugs, and colchicine. The use of steroids is generally avoided as it can precipitate scleroderma renal crisis. Pericardiectomy may be warranted if the disease course is complicated by constrictive pericarditis, which can be life-threatening [14].

## Conclusions

Although evidence of tamponade was only seen on echocardiogram findings in this case, given the high risk of mortality associated with cardiac tamponade, it is important to maintain a high index of suspicion for this complication in scleroderma patients presenting with shortness of breath, even in the absence of hemodynamic instability. The relentless involvement of the pericardium in this case with multiple recurrences suggests an aggressive underlying disease process. More research is needed, but there may be a possible association between anti-RNAP3 seropositivity and cardiac tamponade formation.
